# Cholesterol uptake capacity of HDL in culture medium of fresh primary human hepatocytes: an *in vitro* system for screening anti-atherosclerosis drugs focused on HDL functions

**DOI:** 10.1186/s13104-025-07515-6

**Published:** 2025-11-13

**Authors:** Keishi Hata, Taro Okada, Masaki Takahashi, Yuji Arimatsu, Junko Kawahira, Katsuhiro Murakami, Amane Harada, Nami Yoshikawa, Mutsumi Inamatsu, Masakazu Kakuni

**Affiliations:** 1 Akita Research Institute of Food and Brewing, 4–26 Sanuki, Araya-machi, Akita 010-1623 Japan; 2 Phoenixbio Co., Ltd, 3-4-1 Kagamiyama, Higashi-Hiroshima, Hiroshima 439 − 0046 Japan; 3 Sysmex Corporation, 4-4-4 Takatsukadai, Nishi-ku, Kobe 651–2271 Japan; 4 KMT Hepatech, Inc, 2011-94 Street NW, Edmonton, Alberta T6N 1H1 Canada

**Keywords:** Cholesterol uptake capacity, High-density lipoprotein, Fresh human primary hepatocytes, Eicosapentaenoic acid

## Abstract

**Objective:**

Removing excess cholesterol from atherosclerotic plaques is a crucial function of high-density lipoprotein (HDL). Compared to HDL cholesterol, cholesterol efflux capacity (CEC) is a better indicator of cardiovascular disease risk. However, this approach has several practical disadvantages, such as CEC assay requires cultured cells and takes several days to perform. Recently, we developed a simpler cell-free assay to assess the cholesterol uptake capacity (CUC), a new HDL functionality metric. In this study, we combined the HDL-CUC assay with PXB-cells LA, primary human hepatocytes derived from the humanized mouse liver, to investigate whether the CUC of HDL in the culture medium reflects the eicosapentaenoic acid (EPA) effects on HDL functionality.

**Results:**

The CUC of HDL in the culture medium of PXB-cells LA was measured using the automated immunoassay system HI-1000. Adding EPA to the culture medium did not alter albumin or hepatic triglyceride lipase levels, confirming no significant EPA-induced damage to the hepatocytes. However, as reported for CEC, EPA significantly increased the CUC in a dose-dependent manner, highlighting the potential of EPA as a therapeutic candidate for patients with low CUC. Thus, the proposed assay system could be used for *in* *vitro* drug screening that improves HDL functionality.

## Introduction

In developed countries, metabolic syndrome, caused by a progressive increase in visceral adiposity, has become a serious health problem. In many cases, laboratory animals such as hyperlipidemic mice and rats have been used to screen for antimetabolic syndrome activities; however, laboratory animal studies are less cost- or time-effective than *in* *vitro* studies. We previously reported that PXB-cells, primary human hepatocytes freshly isolated from humanized mouse livers, provide a suitable platform for lipid metabolism research [1]. In addition, PXB-cells LA are available as an *in* *vitro* human non-alcoholic fatty liver disease model [2].

High-density lipoprotein (HDL) cholesterol (HDL-C) is a major traditional risk factor for the prevention and management of cardiovascular diseases [3]. However, some studies have shown that HDL-C-elevating drugs do not reduce cardiovascular events [4, 5]. These findings have drawn attention to evaluating HDL function rather than HDL-C levels. Removing excess cholesterol from lipid-laden macrophages in atherosclerotic plaques for transport to the liver is one of the major physiological functions of HDL, as is the delivery of cholesterol to the adrenals and ovaries for hormone synthesis.

Recent studies have shown that the cholesterol efflux capacity (CEC) of HDL is a better predictor of cardiovascular disease development than HDL-C [6, 7]. However, the CEC assay has practical limitations because it requires cultured macrophages and takes several days. We recently established a novel concept for HDL functionality known as “cholesterol uptake capacity” (CUC), developed an assay system using a fully automated immunoassay device HI-1000, and revealed that CUC correlates well with CEC, acting as a useful predictor of subsequent revascularization in patients undergoing percutaneous coronary intervention (PCI) [8, 9]. In addition, the absolute change in CUC levels from the index PCI to follow-up was lower in patients requiring subsequent revascularization than in those not, suggesting that CUC may be a cardiovascular risk marker and therapeutic target [10]. In the present study, we measured the CUC of HDL derived from the PXB-cells LA to determine whether the CUC of HDL in the culture medium is useful for screening agents that enhance HDL function.

## Materials and methods

### PXB-cells LA and automated HDL-CUC assay

**Fig. 1:**
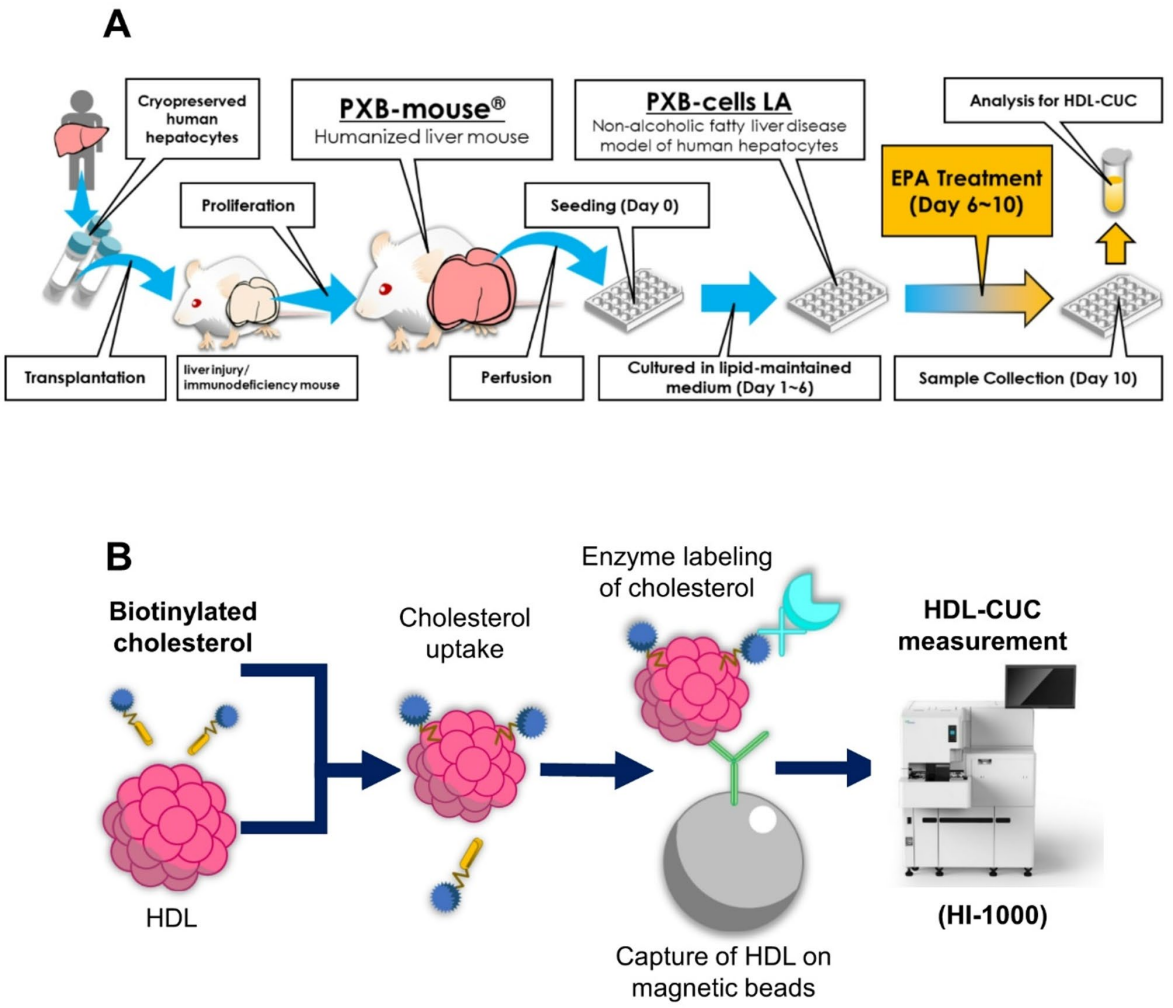
Preparative procedure of PXB-cells LA from PXB-mouse (A) and the principle of HDL-CUC measurement (B)

Fresh human primary hepatocytes (PXB-cells LA) **(**Fig. 1A**)** were isolated from humanized mouse livers 17–18 weeks after transplantation, according to a previously described procedure [2, 11]. PXB-cells LA were seeded at a density of 4 × 10^5^ cells per well in collagen-coated 24-well microplates (denoted as day 0) and cultured in a lipid-containing medium (PhoenixBio Co., Ltd., Higashihiroshima) from days 1 to 6. On day 7, the media was changed to 500 µL of William’s E medium supplemented with CM-4000 (Thermo Fisher Scientific, Waltham, MA, USA) and incubated for 4 days with or without eicosapentaenoic acid (EPA) (Combi-Blocks, Inc., San Diego, CA, USA). The lower exposure level of 0.1 mM (30.2 µg/mL) was referenced from studies using HepG2 cells [12, 13]. A concentration ten times greater was selected as the higher exposure level (302 µg/mL). The higher exposure level was confirmed to be similar to the human plasma exposure level of 366 µg/mL at 5 hours postdosing on day 28, when 4 g of EPA was administered daily [14]. Automated HDL-CUC assays were performed as follows. The culture medium of PXB-cells LA was diluted 1 to 4 in PBS and processed using the method described previously [9, Fig. 1B ] to determine the CUC levels in culture medium.

### ELISA

The levels of albumin, angiotensinogen, hepatic triglyceride lipase, and apolipoprotein A1 (APOA1) in the culture medium were determined with a LZtest “Eiken” U-ALB (Eiken Chemical, Tokyo, Japan), human angiotensinogen and hepatic triglyceride lipase (HTGL) assay kit (Immuno-Biological Laboratories), and an APOA1 ELISA Kit (Abcam) according to the laboratory procedures described in each manual.

### Statistical analysis

Data are expressed as the mean ± standard deviation (SD) of 6 replicates. The significance of the differences between the groups was analyzed using the Kruskal–‒Wallis test, followed by the Steel–Dwass multiple comparison test and the Mann–Whitney U test. Statistical significance was set at *P* < 0.05. Statistical analyses were performed using the Bell Curve for Excel (Social Survey Research Information).

## Results and discussion

**Fig. 2:**
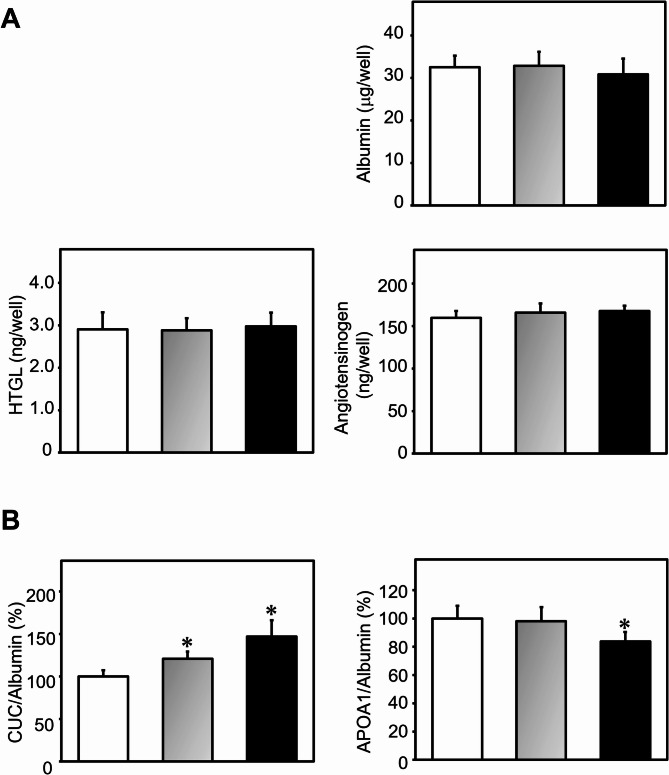
Effects of EPA on several hepatic functions and CUC of HDL in PXB-cells LA

Human studies have shown that the oral administration of EPA, a major polyunsaturated fatty acid in fish, increases HDL efflux capacity, as measured by the dose-dependent efflux of labeled cholesterol from macrophages to HDL in a dose-dependent manner in humans [15]. In this study, we evaluated the effect of EPA on the CUC of HDL composed of APOA1 from PXB-cells LA and examined whether PXB-cells LA derived HDL could be utilized to screen for agents that increase HDL function, as measured by the HDL-CUC assay. Before the HDL-CUC assay, we briefly confirmed whether the two concentrations of EPA affected the hepatic functions of PXB-cells LA by measuring three markers (Fig. 2A). Albumin and HTGL levels have been shown to decrease as liver damage progressed [16, 17]. In contrast, cirrhotic livers retain the capacity to ensure near-normal plasma levels of angiotensinogen until the final stage of liver insufficiency [18]. The present data revealed that EPA at 0.1–1.0 mM did not affect the production of these markers, suggesting that EPA does not cause hepatic dysfunction in PXB-cells LA at these exposure levels.

EPA at 0.1 mM and 1.0 mM markedly increased the CUC activity of HDL in PXB-cells LA culture media by 1.2- and 1.4-fold, respectively, compared with those in untreated cells (left panel in Fig. 2B). To clarify whether the increase in CUC depended on media HDL levels, we measured the levels of APOA1, the main constituent of HDL [19]. EPA at 0.1 mM did not affect the production of APOA1, whereas 1.0 mM suppressed APOA1 production (right panel in Fig. 2B), suggesting that the stimulation of CUC by EPA is independent of media HDL levels. Orally administered EPA is efficiently incorporated into HDL particles, and EPA-rich HDL augments the cholesterol efflux capacity of macrophages without affecting serum HDL-C levels [15]. Thus, our *in vitro* results are consistent with the *in* *vivo* human effects of EPA on HDL CEC activity. Furthermore, measurement of the cholesterol efflux ability of reconstituted HDL consisting of APOA1, cholesterol, and various concentrations of EPA-phosphatidylcholine (EPA-PC) or egg-PC revealed that increasing EPA-PC concentrations increased cholesterol efflux ability [20]. These findings support the results of the present study on the enhancement of CUC by EPA without affecting the HDL levels in the medium. Whole-lipid compositional analysis of HDL secreted from PXB-cells LA treated with EPA is needed to elucidate these events.

Each analysis was performed with 6 replicates. A: PXB-cells LA were incubated with 0.1 mM (gray bar), 1.0 mM (black bar), or no EPA (white bar) for four days, and the levels of extracellular hepatic markers were determined by ELISA. B: CUC (left panel) and APOA1 contents (right panel) in PXB-cells LA treated with 0.1 (gray bars), 1.0 mM (black bars), or without EPA (white bar). 2* *P* < 0.05 vs. without EPA.

Taken together, the results of this study demonstrate that the HDL-CUC assay using the PXB-cells LA culture medium as the source of HDL is a useful tool for screening agents that can improve HDL function. Unfortunately, there is no clinical evidence showing a direct relationship between HDL-CUC levels and cardiovascular disease because this method is new and still in development. However, we consider it is worth investigating pemafibrate via the current HDL-CUC assay and PXB-cells LA, as this medicine increases HDL-C without adverse effects [21] and has been reported to improve HDL-CEC [22]. Furthermore, analysing the effects of classic statins and fibrates on HDL-CUC could support our understanding of the effects of pemafibrate.

The present study revealed the potential of the platform of the HDL-CUC assay combined with PXB-cells LA for the development of HDL functional agents in the future. We are continuing to evaluate this platform by investigating these agents.

## Limitations

Currently, the usefulness of the combined HDL-CUC assay and PXB-cells LA is limited to *in vitro* screening of anti-atherosclerotic activity.

## Data Availability

All the data and alternative results are provided in the manuscript.

## Abbreviations

APO: Apolipoprotein
CEC: Cholesterol efflux capacity
CUC: Cholesterol uptake capacity
EPA: Eicosapentaenoic acid
HDL: High-density lipoprotein
PCI: Percutaneous coronary intervention
